# Artificial intelligence–augmented lesion recognition of peritoneal endometriosis: a novel technique

**DOI:** 10.1016/j.xfre.2026.04.012

**Published:** 2026-05-05

**Authors:** Timur K. Seckin, Hakan Kula, Tamer A. Seckin

**Affiliations:** a Burnett School of Medicine, Texas Christian University, Fort Worth, Texas; b Endometriosis Foundation of America, New York, New York; c Seckin Endometriosis Center, New York, New York; d Department of Gynecology, Lenox Hill Hospital, New York

## Abstract

**Objective:**

To describe a novel surgical approach for the detection and excision of peritoneal endometriosis using intraoperative Aqua Blue Contrast (ABC) with real-time AI-assisted detection.

**Design:**

Case report describing a novel intraoperative surgical technique.

**Subjects:**

A 28-year-old woman presenting with cardinal symptoms of endometriosis.

**Exposure:**

Peritoneal endometriosis presents a significant diagnostic and surgical challenge due to the heterogeneity of lesion subtypes, including pigmented, occult, and microscopic forms. In this approach, intraoperative ABC was applied as a contrast-enhancing technique to improve visualization of the peritoneal surface. In parallel, AI-assisted lesion recognition was used as an adjunctive tool to augment intraoperative recognition of suspicious peritoneal areas. The technique enhances color differentiation, allowing identification of subtle peritoneal abnormalities that may not be easily visible under standard white-light laparoscopy.

**Main Outcome Measures:**

Intraoperative visualization and identification of peritoneal endometriotic lesions.

**Results:**

The ABC enhanced visual contrast between normal and abnormal peritoneal tissue, facilitating identification of occult lesions. The improved visualization with AI-assisted lesion recognition system supported more precise localization and excision of suspected endometriotic areas during surgery.

**Conclusion:**

ABC with real time AI augmentation represents a promising intraoperative visualization technique for improving detection of peritoneal endometriosis. By enhancing lesion visibility, this approach may contribute to more accurate identification and more complete surgical excision.

## CRediT Authorship Contribution Statement

**Timur K. Seckin:** Writing – original draft, Validation, Project administration, Methodology, Investigation, Formal analysis, Data curation. **Hakan Kula:** Writing – original draft, Validation, Software, Resources, Formal analysis, Conceptualization. **Tamer A. Seckin:** Writing – review & editing, Supervision, Methodology, Data curation, Conceptualization.

## Declaration of Interests

T.K.S. has nothing to disclose. H.K. has nothing to disclose. T.A.S. has nothing to disclose.
